# La médecine alternative et complémentaire: quelle place dans le traitement des hémopathies en Afrique?

**Published:** 2012-06-08

**Authors:** Illias Tazi, Hatim Nafil, Lahoucine Mahmal

**Affiliations:** 1 Service d’Hématologie, CHU Mohamed VI, Université Cadi Ayyad, Marrakech, Maroc

**Keywords:** Médecine alternative, Médecine alternative, hémopathies, traitement, Afrique

## Abstract

Lettre aux éditeurs du Journal Pan Africain de Médecine.

## Monsieur le rédacteur en chef du Journal Pan Africain de Médecine

Les hémopathies, quelles soient bénignes ou malignes représentent un véritable problème dans le continent africain. Malgré les grands efforts entrepris par certains pays d’Afrique, la prise en charge de nombreuses maladies du sang demeure toujours insuffisante, par manque de personnel qualifié, par de manque de médicaments adéquats et par des ressources financières très insuffisantes voire même absente dans certaines régions [1]. Les moyens de diagnostic et de traitement ne sont pas suffisamment développés. La majorité des pays d’Afrique préfère allouer leur budget dans d’autres domaines médicaux tel l’exemple de la santé de la mère et de l’enfant. Dans la majorité des pays d’Afrique, les programmes de prévention et de traitement des hémopathies font défaut ou sont insuffisants. La médecine alternative et complémentaire (MAC), peut-elle pallier à ses insuffisances?

Malgré que la MAC ne bénéficie pas d’études scientifiques pertinentes sur son efficacité, ses bénéfices, ses toxicités et ses interactions avec la médecine conventionnelle, elle demeure une partie intégrante de la prise en charge du malade d’hématologie. Elle est souvent reléguée au second plan dans les sociétés occidentales, cette médecine n’a plus de frontières de nos jours dans les pays en voie de développement [2]. Elle peut se baser sur l’utilisation de plantes, de produits minéraux ou animaux [3]. Parfois, le malade a recourt aux pointes de feu, exorcismes, raboutage ou fumigations.

L’hématologie, spécialité médicale de développement récent, est de pratique peu évoluée en Afrique. Le contexte socio-économique, marqué par la pauvreté et l’ignorance, contribue à une prise en charge tardive des hémopathies, découvertes le plus souvent à un stade avancé. On peut se poser toutes les questions qui suivent, car elles demandent toutes une certaine réflexion vis-à vis des MAC: Faut-l opposer médecine moderne et médecine traditionnelle? Peuvent-elles être complémentaires? Y a-t-il une réelle efficacité?

Le recours à ses pratiques est d’autant plus important que les malades sont analphabètes ou issus d’une classe sociale défavorisée. L’usage de traitements complémentaires est un marqueur d’une plus grande détresse psychosociale et d’une plus mauvaise qualité de vie. Les pays en voie d’émergence sont confrontés à des itinéraires thérapeutiques multiples dans la prise en charge des hémopathies. La problématique de la MAC est beaucoup plus importante qu’on ne puisse l’imaginer en hématologie. Son utilisation en cancérologie est estimée entre 18 et 83% [4]. Certains patients utilisent en même temps les deux types de traitement aussi bien conventionnel que traditionnel [5]. D’autres au contraire abandonnent complètement les traitements conventionnels au profit des MAC.

Si l’on se pose la question: quelles sont les raisons de l’attirance pour la MAC? La réponse est certainement difficile à formuler puisqu’elle dépend de nombreux paramètres. Le premier est lié au malade: face à une maladie grave ou chronique et face au besoin réel de guérir, le patient peut associer les traitements prouvés scientifiquement et non prouvés. Le refus du traitement standard et le recours aux remèdes traditionnels peut s’avérer nécessaires selon lui. Le deuxième paramètre à prendre en compte est le paramètre familial: l’entourage du patient peut faire pression pour que l’individu utilise la MAC, cette pression dépend du contexte traditionnel. En effet, l’intérêt majeur de son utilisation est que le malade bénéficie d’une prise en charge optimale afin d’éviter toute culpabilisation ou tout remord s’il y a échec du traitement. La troisième raison de l’utilisation de la MAC est liée au contexte culturel. Le malade peut avoir confiance en remèdes traditionnels conseillés par ses proches avec un refus total de la médecine moderne dont il n’a aucune expérience. Le quatrième point important qui posse le patient à la MAC est la mauvaise communication médecin-malade, où l’échange, la compréhension et la confiance sont pauvres. Beaucoup de médecins ne connaissent par la MAC et doutent de sa réelle efficacité surtout en face d’une maladie grave voire même mortelle.

Nous pensons que la MAC demeure une partie intégrante de la prise en charge du malade en Afrique. Certes elle ne va pas remplacer le traitement indiqué lui-même tel la chimiothérapie ou la radiothérapie mais elle aura un grand rôle dans le soulagement de l’anxiété, dans l’atténuation des symptômes physiques et mal contrôlés.

Nous pensons aussi qu’il est nécessaire aux professionnels de santé de connaitre la MAC afin d’avertir le malade des bénéfices et des risques encourus, surtout la mauvaise compréhension de médecines parallèles ou le malade ne connait pas le but de ce genre de traitement. En hématologie, la nécessité de prendre et de suivre le traitement conventionnel prescrit et indiqué par le praticien est une obligation, la MAC peut se caser au second rang afin de ‘soulager ‘ le patient pour un meilleur suivi. Le débat reste ouvert étant donné que la conception de ces thérapeutiques a évolué avec le temps.

Un autre point important à discuter est la politique sanitaire dans divers pays qui doit permettre au malade de bénéficier des soins et de services dans toutes les régions du continent même pour les plus démunis d’entre eux. Cette disposition devrait faciliter l’itinéraire thérapeutique pour le malade pour un meilleur respect du choix du malade, pour une meilleure aide et surtout pour un meilleur accompagnement.

En conclusion, la MAC peut parfois séduire les malades au point qu’elle peut les priver du traitement utile scientifiquement établi. C’est redire tout l’intérêt d’une communication de bonne qualité, d’une ouverture d’esprit et d’une disponibilité optimale des praticiens pour une prise en charge optimale des malades atteints d’hémopathies dans le continent africain.

## References

[1] Tazi I, Nafil H, Mahmal L (2011). Palliative care in hematology: what future in Africa?. Pan Afr Med J..

[2] De Smet PA (2002). Herbal remedies. N Engl J Med..

[3] Stewart MJ, Steenkamp V, Zuckerman M (1998). The toxicology of African herbal remedies. Ther Drug Monit..

[4] Sparber A, Bauer L, Curt G, Eisenberg D, Levin T, Parks S (2000). Use of complementary medicine by adult patients participating in cancer clinical trials. Oncol Nurs Forum..

[5] Richardson MA, Sanders T, Palmer JL, Greisinger A, Singletary SE (2000). Complementary/alternative medicine use in a comprehensive cancer center and the implications for oncology. J Clin Oncol..

